# Natalizumab in Multiple Sclerosis Treatment: From Biological Effects to Immune Monitoring

**DOI:** 10.3389/fimmu.2020.549842

**Published:** 2020-09-24

**Authors:** Kathy Khoy, Delphine Mariotte, Gilles Defer, Gautier Petit, Olivier Toutirais, Brigitte Le Mauff

**Affiliations:** ^1^Laboratory of Immunology, Department of Biology, CHU Caen Normandie, Caen, France; ^2^Department of Neurology, MS Expert Centre, CHU Caen Normandie, Caen, France; ^3^UMR-S1237, Physiopathology and Imaging of Neurological Disorders, INSERM, Caen, France; ^4^Normandie Université, UNICAEN, Caen, France

**Keywords:** multiple sclerosis, natalizumab, biotherapy, drug modifying therapy, Mab therapy monitoring, Integrin, neutralizing antibodies, PML

## Abstract

Multiple sclerosis is a chronic demyelinating disease of the central nervous system (CNS) with an autoimmune component. Among the recent disease-modifying treatments available, Natalizumab, a monoclonal antibody directed against the alpha chain of the VLA-4 integrin (CD49d), is a potent inhibitor of cell migration toward the tissues including CNS. It potently reduces relapses and active brain lesions in the relapsing remitting form of the disease. However, it has also been associated with a severe infectious complication, the progressive multifocal leukoencephalitis (PML). Using the standard protocol with an injection every 4 weeks it has been shown by a close monitoring of the drug that trough levels soon reach a plateau with an almost saturation of the target cell receptor as well as a down modulation of this receptor. In this review, mechanisms of action involved in therapeutic efficacy as well as in PML risk will be discussed. Furthermore the interest of a biological monitoring that may be helpful to rapidly adapt treatment is presented. Indeed, development of anti-NAT antibodies, although sometimes unapparent, can be detected indirectly by normalization of CD49d expression on circulating mononuclear cells and might require to switch to another drug. On the other hand a stable modulation of CD49d expression might be useful to follow the circulating NAT levels and apply an extended interval dose scheme that could contribute to limiting the risk of PML.

## Introduction

Multiple sclerosis (MS) is a chronic, inflammatory autoimmune disease leading to demyelination. It is a heterogeneous, multifactor disease with environment factors acting in a susceptibility genetic background, still only partially described. Following a silent phase, the most common clinical form of MS is the relapsing remitting MS (RRMS) with accumulation of lesions during relapse phases. With time the disease may evolve as a progressive phase without remission (secondary progressive MS, SPMS) although some patients may have a progressive disease from the onset called primary progressive MS (1). Although few treatments are active on the progressive forms of MS, the treatment of RRMS has been dramatically modified in the era of monoclonal antibodies and other disease modifying therapies (DMT).

Among them, Natalizumab (Tysabri^®^, NAT) is a humanized IgG4 antibody (Ab) that recognizes α4 chain (CD49d) of the VLA4 (Very Late Antigen 4) antigen, a component of the α4β1 integrin, and of the α4β7 integrin. It is the clinical achievement of the pioneer work of Yednok et al., who demonstrated the role of this adhesion molecule in the interaction of leukocytes with inflamed endothelium in the brain and had shown that the injection of an anti-α4 monoclonal antibody prevents EAE (experimental autoimmune encephalomyelitis) in a rat MS model (2). Consequently, a mouse anti-human α4 chain Ab able to block VLA4 interaction with its ligand VCAM1 (Vascular Cellular Adhesion Molecule1) was selected for humanization (3). Two phase III studies demonstrated the efficacy of NAT to improve the evolution of RRMS in terms of annual relapses or development of brain MRI lesions (4, 5). This success was not obtained in SPMS (6). In addition, a severe adverse effect was then reported with the appearance of progressive multifocal encephalopathy (PML) (7) which was usually occurring in immuno-compromised or immunosuppressed patients. The standardized protocol consists in a 300 mg dose every 4 weeks but many schemes extending interval dosing have been tested with similar efficacy (8–11).

## Mechanisms of Action

The underlying MS pathological process involves both antigen specific and non specific inflammatory mechanisms. Part of the knowledge is coming from animal studies using the EAE model (12) but contradictory features concerning the human pathology have emerged from several therapeutic trials. For example, the central role of antigen specific T cells observed in EAE has been extended from CD4+ T cells in EAE to more numerous CD8+ T cells in human with autoreactivity against myelin derived peptides, and from a critical role of Th1 cells secreting IFNγ to the participation of Th17 cells producing GM-CSF (13, 14). The importance of B cells has long been recognized with the presence of oligoclonal bands in CSF, but the recent evidence of the efficacy of therapies depleting these cells without significant effects on immunoglobulins shifts their role toward their ability to present antigens to T cells (15). Furthermore a complex inflammatory infiltrate in central nervous system (CNS) and CSF is described including various innate cells that complete the role of the locally activated microglia. Whatever the mechanisms, the activation of antigen specific lymphocytes either in the periphery or not, and the secondary colonization of CNS need cell interactions and migration which are dependent on chemokines and adhesion molecules. The rationale for using anti-α4 Abs in EAE was their blocking effect on the adhesion of leukocytes leading to inhibition of inflammatory migration to CNS. Although a large body of results strengthen this strategy, some pre-clinical data suggest that according to the timing of monoclonal Ab (Mab) administration or the experimental model, anti-α4 Abs can be inefficient or deleterious despite VLA4 blockade (16, 17), possibly because of agonistic properties of anti-VLA4 Abs (17). Nevertheless the activating effect of anti-VLA4 Abs has not been described in NAT treated patients (18–20). In addition, in EAE models, infiltration of Th17 cells or GM-CSF-producing Th1/Th17 cells into the CNS has been shown to be mediated by lymphocyte function associated antigen 1 (LFA1) adhesion molecules and not VLA4 integrin, thereby suggesting more differential effects of anti-VLA4 blockade, at least, in animal models (21, 22).

By preventing the interaction of α4β1 integrin expressed on lymphocytes to its ligand VCAM1 on endothelial cells, NAT inhibits the migration through the brain blood barrier into the CNS parenchyma. There are two ways to confirm this effect: in the blood compartment, an increase of leukocytes has already been observed (23) whereas a decrease of infiltrating cells could be assessed in the CSF. Evidently, CNS infiltrating lymphocytes were decreased in patients treated with NAT as compared to untreated patients or pre-treatment levels. This was observed for T lymphocytes, mainly for CD4+ cells, and for B cells (24–28) and led to a diminished level of immunoglobulins (IgM, IgG) including oligoclonal bands, with a decrease of local production (24, 27–29). These effects were confirmed in longitudinal studies and disappeared – albeit slowly (within 6 months) – after treatment interruption (26). Monocytes were increased relatively to lymphocytes during treatment suggesting that their migration might be less VLA4-dependent (30, 31). Few reports analyzed the effects of NAT on antigen presenting cells, but a reduced number of dendritic cells (DCs) had been observed in perivascular spaces in post mortem samples of a NAT treated patient (32). Furthermore, in addition to a decreased expression of CD49d, both myeloid and plasmacytoid DCs had impaired capacities to stimulate T lymphocytes (33).

As a consequence of this extravasation blockade, mononuclear cells accumulate in the circulation. In addition, some haematopoietic precursors might be released from the bone marrow due to loss of VLA4-VCAM1 interactions with the stromal cells or altered homing (34, 35). The net result is an important lymphocytosis following the first injection which soon reached a stable plateau. The more altered cells were B lymphocytes (more than 3 times pre-treatment values), NK and T lymphocytes (2 and 1.8, respectively) without modification of the CD4+/CD8+ ratio (36–38). Cell numbers decreased after 8 weeks of treatment interruption and returned to basal levels around 16 weeks after this interruption (38). The phenotype and function of the circulating cells have been explored and inconstantly showed an increase of memory T cells which might reflect their higher CD49d expression, and of activated cells (18, 39, 40). Although Th17 or Th1/17 cell migration has been suggested to be partially VLA-4 dependent (31), it is mostly observed that under NAT treatment these cells also accumulate in the circulation (41, 42). Furthermore, the frequencies or proliferative capacities of potential encephalogenic myelin basic protein reactive cells were not modified under NAT treatment (39). Some variations in cytokine production merely pro-inflammatory were also observed, especially in the early phases of treatment (39, 43, 44). In contrast, no quantitative nor qualitative effect was noted on regulatory T cells (Tregs) (18, 45). These cells constantly showed a strong decrease of CD49d expression (46, 47) but their migration was still efficiently blocked and their suppressive effects preserved (47). B cells were the most impacted circulating cells and also demonstrated a memory phenotype, prone to activation, and pro-inflammatory profile (25, 40, 48).

A direct activation role of natalizumab through CD49d has been excluded for all types of cells (25, 39) arguing for a mere accumulation in the circulation of cells potentially activated, due to the inhibition of migration. It might favor the recurrence of the disease after treatment interruption, observed in approximately one third of the cases, which needs a switch to another treatment (49). In some cases a more severe relapse is observed as compared to the pre-treatment status of the patient, described as a rebound effect (50) and can be related to the migration of autoreactive Th1, Th17, or Th1/17 cells accumulated in the circulation during NAT treatment (41, 42).

## PML Complication

Progressive multifocal encephalopathy, a demyelinating disease caused by the John Cunningham virus (JCV), was soon observed in NAT treated patients although it was previously associated with immunodeficiency or immunosuppression (7). Despite a high incidence (1/1000) with an 18-month treatment, (51), a clear benefit/risk balance reinstated it after a short market withdrawal. In MS treatment, other drugs such as anti-B cell Mabs (anti-CD20 Mabs), dimethyl fumarate, or fingolimod had an increase of PML risk, but far less than NAT (52). Another anti-adhesion molecule, efalizumab (anti-LFA-1) used in psoriasis, has been withdrawn because of PML complications (53). The concept of altered immune surveillance to virus in CNS due to the cell circulation migration inhibition has long been the main argument described as the cause of this increased risk. However, some properties of NAT might facilitate this disease. The JCV infection is a very frequent asymptomatic disease usually occurring during childhood, then remaining latent until a possible reactivation, which remains a very rare event. Although the knowledge of JCV biology has greatly improved, some critical issues persist about the process of latency and reactivation (54). It has been suggested that the increase of circulating haematopoietic precursors and/or the accumulation of pre-B and B cells (34, 35, 55–58) might represent a potential virus reservoir for JCV (59, 60). Analysis of JCV in these cells showed some conflicting results (35, 60–63), probably depending on method sensitivity. Nevertheless, when detectable, it should be mentioned that the virus is detected at low levels or under inactive form; and sometimes in asymptomatic patients (60, 61, 64). These data are consistent with a latency phase of the virus. In addition, normal brain might also be another site of latent viral persistence (65).

It has been shown that NAT is able to upregulate transcription regulators POU2AF1 and Spi-B in B cells (59, 66). Consequently, transition from latent archetype to prototype virus variant, viral transcription and replication are suspected to be facilitated in lymphoid cells (60, 62, 67). Spreading to CNS through B cells or free virions is speculated but has not been proven (68). But, even if this hypothesis is true in immunocompetent people, it is likely that the spreading would be inhibited under NAT treatment. On the target cell side, NAT has not been shown to facilitate neural cell infection, at least *in vitro* (69). In the context of immune modulation induced by NAT, there is a decrease in antigen presenting cells in the CNS (32), and the trafficking of memory T cells is not selectively inhibited by NAT. It has also been shown that the anti-viral Th1 compartment is retained in the circulation hampering the JCV elimination (41). At this stage, the main parameters for susceptibility to JCV infection are NAT treatment longer than 2 years, prior immunosuppression and anti-JCV seropositivity.

## Drug Monitoring

### Circulating and CSF Levels of NAT

As for most drugs, the measurement of concentrations is a tool to determine the best dosage. Various methods have been used to measure NAT concentrations. Due to its heterodimeric structure, cellular assays have been developed using cells expressing CD49d and FACS analysis with a standard curve of NAT (70, 71). Alternatively ELISA methods have been set up. A particular property of the IgG4 isotype that has been uncovered is that due to the absence of covalent links between the two heavy chains, “Fab arm exchange” occurs between IgG4, rendering them monovalent (72). In addition to potentially modifying NAT functional effect, it can directly interfere with detection assays. Accordingly, an alternative to classical bridging test has been developed (73) but no strict comparison measurements have been thoroughly published yet. The variable median results of NAT free circulating levels observed among studies (from 18 to 51 μg/ml) may be assay dependent, but a common characteristics noted within each study was the high variability among patients (less than 4 μg and up to 100 or 200 μg) (71, 74, 75). No clear relationship has been evidenced to identify factors involved in this heterogeneity although body weight might contribute (76, 77). Nevertheless, for a given patient, trough levels soon reach a plateau and remain stable whatever the number of infusions (9) and for more than 90% of them were over 10 μg/ml (78). In comparison, levels within CSF were a hundred times lower from 45 to 110 ng/ml (71, 74).

In the serum, free NAT was measured, but the cell bound part can also be determined. Cytometry allows determining the level of NAT bound to cells using a fluorescent anti-IgG4 antibody, as well as the free CD49d molecules on the cells that are not covered by the administered drug, using an additional incubation with an excess of NAT. This assay is suitable for determining the saturation level of CD49d on the cells which, although slightly different according to the circulating cell type analyzed, is around 70% (79, 80). Surprisingly, and despite the low levels of free NAT measured in CSF, nearly the same degree of saturation was observed in CSF (79).

These assays were performed during ongoing treatment but the disappearance of NAT was also evaluated in studies performed after interruption of treatment (38, 81). In the RESTORE study designed to evaluate the consequences of treatment interruption, NAT circulating levels after the last injection differed from patients still treated 8 weeks after interruption of treatment, and it takes 16 weeks for the NAT levels to become undetectable (38). In parallel, at the same time, the saturation of circulating cells started to decrease (68% vs 87% for treated patients) but some antibody remains detectable on the cells between 16 and 28 weeks after interruption (38).

When the clearance of NAT needs to be very rapid, for instance because of PML, protocols of plasma exchange are used and allows almost 90% elimination of circulating NAT within 1 week. In these conditions, the saturation of the cells falls under 50% when NAT is <1 μg/ml, and partial restoration of migratory capacities is obtained 3 weeks after plasma exchange treatment (82). It should be mentioned that this strategy is not without risk. In addition to a potential reactivation of the disease, it may represent a worsening factor in PML, inducing an immune reconstitution inflammatory syndrome (IRIS) that leads to a poorer prognosis that in case of spontaneous NAT clearance (83).

### Pharmacodynamic Analysis

These pharmacokinetics parameters have been completed by pharmacodynamic analysis checking some dose-dependent functional effect. Parallel to the receptor saturation, it could be noticed that CD49d expression, as determined using a fluorescent anti-CD49d antibody recognizing another epitope, was decreased around 50% of the pre-treatment level soon after treatment initiation (19, 70, 71, 84). It then remained stable all along treatment except in cases of immunization (cf infra). This diminished expression, associated with a decrease of CD29, the β1 chain of this heterodimer, (84) might contribute to the inhibition of VLA4/VCAM interactions. The recovery of the expression after treatment interruption is slower than the decrease of receptor occupancy (9).

Using fluorescent beads allowing quantification (Quantibrite, BD), a more precise evaluation has been performed to compare the number of membrane expressed CD49d molecules and the number of bound NAT molecules (85). It allows a direct estimation of the level of saturation in patients receiving standard protocol (Standard interval dosing SID, 4 weeks) or protocols with an extended interval (EID) between two injections. This schedule was evaluated in order to limit the risk of PML. Using a regular treatment, T CD4, CD8, B cells expressed, according to the cell type, around 1300–1400 CD49d molecules. In contrast with an interval of 6 weeks between injections, the number of CD49d was 2000–2400 molecules/cell. Nevertheless, the number of NAT bound molecules was not different between the 2 groups leading to decreased receptor occupancy (RO) from 76–84% to 54–62% (85). Using a simple measurement of the mean fluorescence intensity of an anti-CD49d antibody, a modest increase of CD49d expression was observed in EID (9%) as compared to SID, still at 60% of the pre-treatment levels, and it was associated with a decrease of NAT circulating levels from 36 to 18 μg/ml (9). These trough levels are still over the levels needed for an almost receptor saturation. With these EID protocols, no worsening of the clinical status was noticed suggesting that increasing the time between injections is not altering efficacy (10, 11).

So, biological parameters for monitoring the interval injection duration are available. As far as now, no studies have determined a critical level for saturation or modulation of CD49d required for clinical efficacy. These parameters might be useful for an adaptation of dose or timing on a case by case basis to limit the adverse biological effects of NAT.

### Anti-drug Antibodies

Therapeutic strategies were greatly completed by introducing monoclonal antibodies but despite the molecular engineering of humanized molecules these proteins keep a potential immunogenicity especially when used as monotherapy. In the case of NAT, nearly 9% of the patients were identified with anti-NAT antibodies, and 6% are immunized permanently (4). For some patients the injection related side effects suggest immunization, that needs to be investigated, whereas for many of them the process is silent or relapses might occur by therapy inhibition. For these patients, a systematic screening for immunization has been suggested at 6 months. The presence of high titers of anti-NAT antibodies is suggestive of a permanent immunization. Depending on the test used, no clear cut-off has yet been defined (75, 86, 87). However, in our experience, transient anti-NAT Ab were detected at rather low levels (10 times less) as compared to patients with persistent neutralizing antibodies (70). The neutralizing effect of immunization can also be suggested by using the monitoring parameters previously discussed. Among them, the end of CD49d expression down-modulation is suggestive of the immunization (70) which can be either transient or permanent.

Immunization is also responsible of NAT clearance, and complete disappearance of circulating free NAT was observed in immunized patients with clinical relapse (75). Depending on the local laboratory practice, it can be easier and more flexible to measure modulation of CD49d for a given patient than to perform complete series of natalizumab and anti-natalizumab ELISA. The measurement of the lymphocytosis has also been suggested to be a potential biomarker of efficacy (88) but has not been related to NAT levels, saturation, or anti-NAT antibody appearance.

In-depth analysis of the immune response of two patients has allowed the characterisation of the B and T cell responses. In contrast to the large polyclonal anti-idiotypic B response, an immunodominant T cell epitope was identified in the FR2-CDR2 region of NAT light chain. In addition this epitope could be modified to avoid T cell recognition without loosing the binding to CD49d (89) providing a deimmunized antibody (90). Such a modified molecule could be an alternative for immunized patients.

In conclusion NAT is one of the recent therapies that have changed the evolution of RRMS. However, long term treatment has been associated with PML, a severe infectious complication. No specific biologic risk linked to NAT properties has been definitively identified in this susceptibility, which is also observed in other immunosuppression states either related to HIV or monoclonal antibody treatments or other DMT. In the context of NAT, no drug overdose was noticed at the time of infection (77) and risk evaluation remained to be assessed on treatment duration and anti JC antibody status. In order to limit the risk of PML, EID protocols seem to maintain a sufficient efficacy, although the real benefit on large cohorts has not yet been reported, and the ongoing NOVA study might contribute to this evaluation (91). On the other side, inefficient treatment might not always be clinically detectable until new release. In both circumstances, to offer an optimized treatment with potential therapeutic switch and to improve the cost/benefit, it might be interesting to develop an adapted biological monitoring using an easy-to-measure parameter such as modulation of the expression of CD49d, which is a good and robust functional reflect of the circulating levels of NAT.

## Author Contributions

All authors contributed to manuscript revision, read and approved the submitted version.

## Conflict of Interest

The authors declare that the research was conducted in the absence of any commercial or financial relationships that could be construed as a potential conflict of interest.
